# Escape our Lab: creating an escape room game in the field of materials science and crystallography

**DOI:** 10.1107/S1600576723006714

**Published:** 2023-08-18

**Authors:** Christian Schimpf, Jonas Lachmann, Marius H. Wetzel, Peter D. B. Fischer, Andreas Leineweber, David Rafaja

**Affiliations:** a Institute of Materials Science, TU Bergakademie Freiberg, G.-Zeuner-Straße 5, Freiberg 09599, Germany; Wilfrid Laurier University, Waterloo, Ontario, Canada

**Keywords:** escape rooms, STEM education

## Abstract

Although many challenges of the 21st century need solutions which are directly connected with the development of new technologies, the preferences of prospective students in Germany are often far from mathematics, physics and chemistry. Moreover, the acceptance and recognition of new achievements in these disciplines are quite low in society, even if these achievements are the basis for the development of new technologies that positively affect daily life. As a part of a campaign intended to increase the number of students in the fields of materials science and materials technology (and related fields), the authors created an escape room focused on materials science and crystallography, which illustrates the approaches used by materials scientists and the beauty of crystallography. The fundamental features of the escape room, which are presented in this contribution, are its variability and the ability to inspire participants who have different backgrounds in physics, chemistry and/or materials science. By varying the level of difficulty and the game play duration, the escape room structure makes it possible to appeal to a broad audience, offer an authentic escape room experience and impart lasting knowledge through reflection after completion. The authors’ experiences with the escape room and the feedback from the attendees are summarized at the end of the contribution.

## Abstract 2

A variable educational exit game is presented to stimulate interest of young people in STEM-related fields. The game addresses scientific content from crystallography, materials science and related fields and is suitable for a wide range of player groups, such as secondary school pupils, undergraduate students and teachers.

## 1. Introduction

The transformation of society towards environmental sustainability, which requires reduction of greenhouse gas emissions, utilization of renewable energy sources and usage of secondary raw materials, directly implies an increasing demand for specialists in materials science and materials technology. These disciplines belong to the fields of science, technology, engineering and mathematics (STEM), which are currently not in the focus of many prospective students. Despite public awareness of the current situation and many political movements, the interest in this field from young people is on a downtrend (Federal Ministry of Education and Research, 2022 ▸). In Germany, this trend is fortified by the demographic situation, as presently the cohorts with a low birth rate enter universities and the job market (Köller, 2022 ▸). Consequently, a lack of STEM-qualified personnel is expected for the near future (IW Köln, 2022 ▸). To increase the interest of young people in STEM studies, universities have to utilize unconventional methods to attract students. One approach that we have explored is to build an escape room to motivate pupils to study a STEM subject.

Game-based learning is a well established educational concept. New interest in this field has been stimulated by the transformation of recreational escape rooms into an educational setting. Veldkamp *et al.* (2020 ▸) described escape rooms in general as live-action team-based games in which players encounter challenges in order to complete a mission in a limited amount of time and in a confined space. The activities inside an escape room are called ‘puzzles’ and follow a simple systematic sequence: facing a challenge, finding solutions for the tasks and receiving a reward (Veldkamp *et al.*, 2021 ▸). The challenge is, in most cases, to find the correct code for a combination lock. The solution must be found by the players through application of existing or acquiring new knowledge or using simple logic. The reward is usually some object to advance in the game. Escape rooms are suitable teaching concepts for all fields of science (Veldkamp *et al.*, 2021 ▸) and STEM-related educational escape rooms are numerous and quite diversified. Still, review studies (Veldkamp *et al.*, 2020 ▸; Lathwesen & Belova, 2021 ▸) have shown that most escape rooms for higher education were used in the field of medicine and nursing.

A recent review on STEM-related escape rooms (Lathwesen & Belova, 2021 ▸) revealed that such rooms are mainly focused on chemistry (*e.g.* Dietrich, 2018 ▸; Peleg *et al.*, 2019 ▸; Ferreiro-González *et al.*, 2019 ▸), physics (*e.g.*Vörös & Sárközi, 2017 ▸; Sárközi *et al.*, 2019 ▸) and mathematics (*e.g.* Fuentes-Cabrera *et al.*, 2020 ▸; Ho, 2018 ▸). Some examples were also found for escape rooms devoted to informatics (López-Pernas *et al.*, 2019 ▸), astronomy (Yllana-Prieto *et al.*, 2021 ▸) and biology (Healy, 2019 ▸), among others; see references given by Lathwesen & Belova (2021 ▸). No report of an escape room based on materials science or crystallography was found in a survey of existing literature. The escape rooms are often located in schools or at universities. Different concepts for the design and setup of escape rooms are compared by Veldkamp *et al.* (2020 ▸), who found that sequential game paths are prevailing (Lathwesen & Belova, 2021 ▸). Their main purpose is the fostering of knowledge already obtained while focusing on a specific problem within the scientific field. According to Lathwesen & Belova (2021 ▸), the main drawbacks of current educational escape rooms are missing interdisciplinary settings, difficult adaption and transfer to other institutions, overly specific design principles for educational escape rooms (Veldkamp *et al.*, 2020 ▸), and a lack of empirical evidence for the impact of educational escape rooms.

In several studies (Veldkamp *et al.*, 2020 ▸, 2021 ▸; Lathwesen & Belova, 2021 ▸), escape rooms were found to be very effective for enhancing teamwork, increasing motivation on a given subject, and processing and fostering content knowledge (Veldkamp *et al.*, 2021 ▸). Critical thinking is significantly raised in an escape room compared with normal lesson environments (Veldkamp *et al.*, 2021 ▸). However, they are less suited to learning new concepts due to the lack of tranquillity and opportunity for reflection resulting from time pressure for completion, which may easily increase the level of frustration (Veldkamp *et al.*, 2021 ▸). Still, the limited amount of time can also motivate players to engage in mutual interactions and more creative thinking (Veldkamp *et al.*, 2021 ▸). In other words, the development of the four Cs [critical thinking, collaboration, creativity and communication (Breakout EDU 2022, https://www.breakoutedu.com/; van Roekel, 2012 ▸)] seems to be perfectly addressed by escape rooms. However, the perception of the relevance of escape room features between players (students) and creators (teachers) differs. While students found the puzzles themselves, teamwork and the authenticity of the setting most appealing, teachers regarded the competition, winning the prize or the excitement as most attractive to students (Veldkamp *et al.*, 2021 ▸). Finally, time for debriefing or a recapitulation of the experiences seems to be very important for a successful escape room experience for the players.

We developed our escape room for informal education, *i.e.* mainly to excite interest in phenomena previously unknown or underrated by the players. Our intention is to stimulate interest for STEM subjects in general, but for materials science and crystallography in particular. Mainly pupils from high school, but also undergraduate students, have participated in our escape room. In the following, we present a walk-through of our escape room to show how we have set up a flexible escape room concept to address different levels of knowledge of the players. Furthermore, our setup offers the possibility to convey new topics to players or apply present knowledge in a completely new environment. By the nature of materials science, the setting is interdisciplinary and combines elements from physics, chemistry and mathematics. We present a variety of puzzles related to materials science and crystallography and thereby provide a broad insight into these disciplines for the players. Groups of up to eight members can be accommodated in a single game run, which is larger than for most other educational escape rooms (Veldkamp *et al.*, 2020 ▸; Lathwesen & Belova, 2021 ▸).

## 2. A walk through our escape room

**Figure 1:**
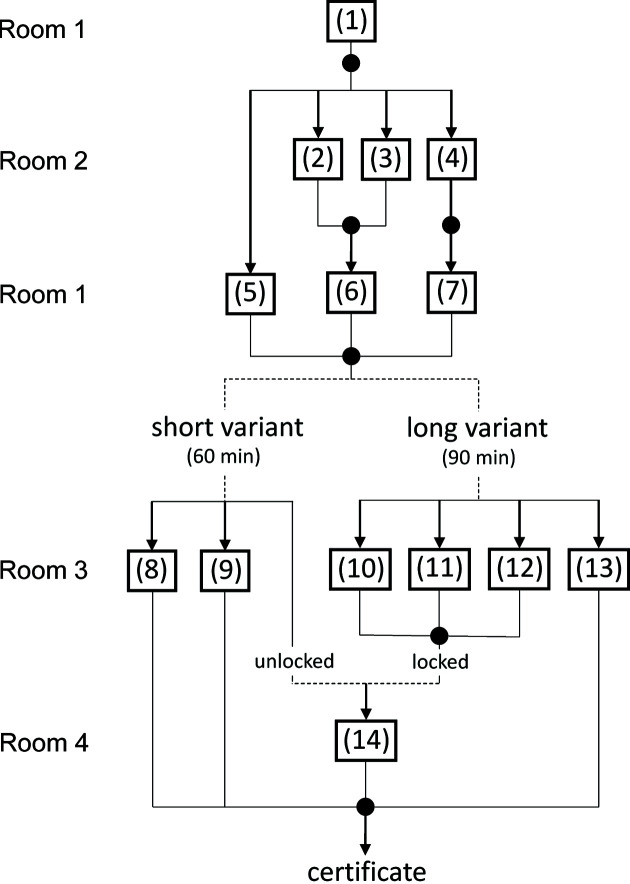
Scheme of the puzzle sequence used in our escape room. Numbers in parentheses correspond to the numbers of the puzzles used in the text. Black dots mark the positions of the combination locks or lock boxes to be opened. The scientific phenomena addressed in these puzzles and the materials used are compiled in Table 1 ▸.

**Figure 2:**
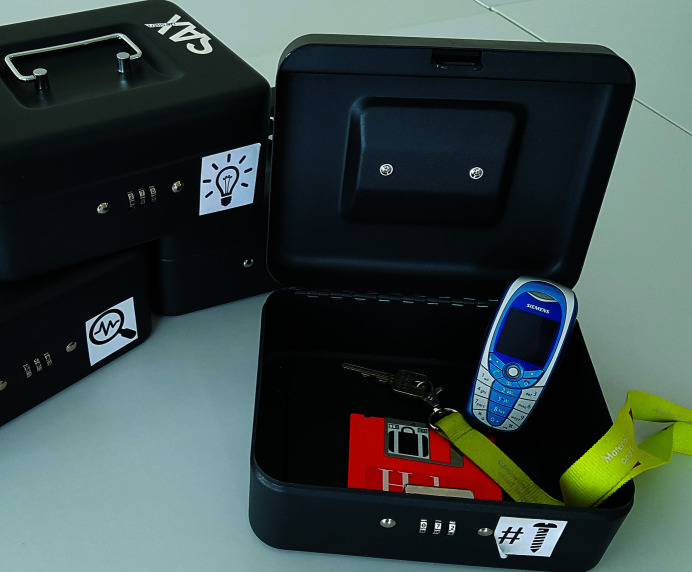
Combination lock boxes used for the escape room, shown here with some items used in the escape room (key to open a door, 3.5′′ floppy disc and mobile phone). Boxes are marked with symbols to highlight to which puzzle they belong.

The setup of our escape room is a combination of ‘path-based’ and ‘sequential’ types (Veldkamp *et al.*, 2020 ▸). A flow chart of our setup is given in Fig. 1 ▸. This approach offers substantial variability regarding the time and proficiency required as well as the number of players that can be engaged. The numbers in Fig. 1 ▸ relate to the puzzles that must be solved by the players. The puzzles themselves are explained below. Each puzzle setting is provided with a note containing hints for its solution. The hints also contain a short explanation of the scientific background of the puzzle. Depending on whether hints are provided to the players or not and how much information is contained in them, the additional hints create different levels of difficulty that match and build on the players’ prior knowledge. The black dots in Fig. 1 ▸ mark the positions of lock boxes or combination locks that the players must open by solving the puzzles to advance the game. Typical lock boxes that we use to store items are shown in Fig. 2 ▸. Table 1 ▸ contains the theme and list of materials for each of the puzzles as well as some remarks on the costs of the materials required.

The central idea of the escape room is that the players find a hastily abandoned laboratory with unfinished experiments related to materials science and crystallography which they should complete. Once all experiments are finished, the players will find the reason why the scientists have left. The experiments are designed as (and hereafter termed) puzzles. The escape room is located in areas that are normally used for laboratory courses and consist of four sub-rooms, which have to be opened sequentially. The available devices and apparatus give the rooms the feeling of ‘real labs’. Authenticity is usually positively recognized by the players (Veldkamp *et al.*, 2021 ▸). Present devices which are not involved in the puzzles of the escape room are marked accordingly.

The escape room game can be performed in a 60 min ‘short’ variant and a 90 min ‘long’ variant. In both cases, the goal is to pass through the whole setting. A strict time-out is not scheduled, as it would destroy the educational concept of the escape room and may evoke frustration among the players as the story would be unfinished. Nevertheless, to establish some time pressure, we confront the players with a time-based ranking list of previous groups at the beginning, which is usually challenge enough for the players and noticeably increases their motivation. The optimal size of a group for our escape game is 6 ± 2 players, in agreement with Veldkamp *et al.* (2020 ▸). Usually one or two ‘game masters’ are present in the escape room with the players for mastering, monitoring and guiding (if necessary) and for safety reasons. The presence of an authority does not degrade the authenticity experienced by the users (Veldkamp *et al.*, 2020 ▸) and is common in educational escape rooms.

### 2.1. Room 1

Before the game starts, the players receive safety instructions and a ‘to-do list’ showing the names of the puzzles and their order (see supporting information). The starting room 1, which is not locked, resembles a laboratory. The laboratory design shows that the experiments are the basis for the work of materials scientists. In our case, it is a thermal treatment laboratory containing furnaces, lab desks, tools *etc*.

**Puzzle (1)**

Determine the number of screws in a bottle. The number of screws is the code to unlock the box in which a key to the next room is retained.

**Figure 3:**
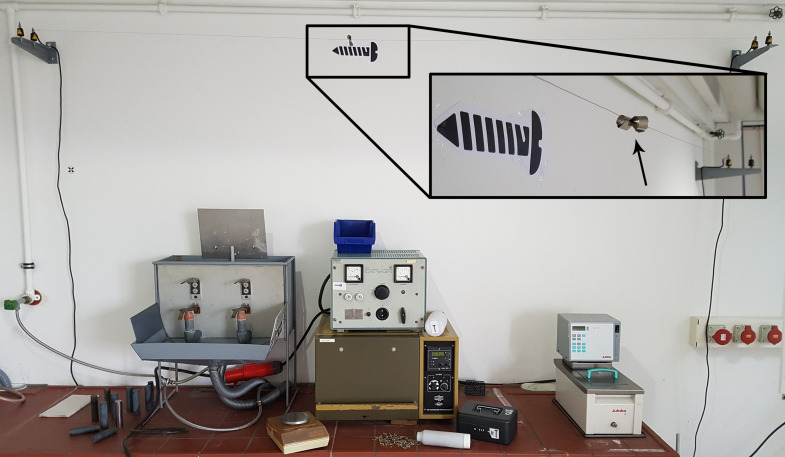
Utilities for puzzle (1): screws in a bottle, scale, power supply, nickel wire, magnet and metal foil with the hint.

*Utilities*: screws, a scale, a calculator, nickel wire, a magnet and a power supply (Fig. 3 ▸).

*Additional hint*: average screw weight written on a metal foil, which is magnetically attached to an Ni wire mounted underneath the ceiling (inset in Fig. 3 ▸).

The goal for this room is to solve puzzle (1) (see Fig. 1 ▸) and open a lock box, which contains the key to room 2. The code for unlocking the box (a three-digit number) is equal to the number of small screws in a bottle which can be found in room 1. The number of screws is quite large. Thus, it would be extremely time consuming to count them. Another option is to determine the number of screws from their total weight divided by the average mass of a single screw. A scale is provided. Still, it must be considered that the masses of individual screws can differ by ±10% from each other. Thus, attempts to determine the average mass by weighing only a few screws fail in most cases due to the large fluctuation of the individual weights of the screws. Therefore, we provide the average weight of a single screw as an additional hint, which is located below the ceiling of room 1 and can only be accessed by solving a puzzle.

A small piece of a metal foil with the hint written on it is attached by a permanent magnet to a wire made from Ni. At room temperature, Ni is ferromagnetic, which is why the permanent magnet sticks to the wire. Above 358°C, which is the Curie temperature of Ni, the wire becomes paramagnetic. Above this temperature, the permanent magnet attached to the wire falls down and releases the metal foil. For heating up the Ni wire, Joule heating is utilized, produced by the electrical current supplied by the available power supply. The whole setup is shown in Fig. 3 ▸. The power supply is located in the middle of the photograph. The metal foil with the hint is shown in detail in the insert. After the players have correctly determined the number of screws in the bottle, they can open the lock box. It contains the key to open room 2 and a 3.5′′ floppy disc, which will be used later.

### 2.2. Room 2

Room 2 is a seminar room that is furnished with a chalkboard, tables and chairs. The change from a laboratory to a seminar room illustrates the second facet of the work of materials scientists, which is the evaluation of the experimental data and the interpretation of results based on theory. In this room, several puzzles connected to crystallography must be solved to open another two lock boxes. To open the first lock box, puzzles (2) and (3) (*cf*. Fig. 1 ▸) must be solved in order to obtain the first three-digit code. Puzzle (4) must be solved to open the second lock box.

**Puzzle (2)**

Count the rotation axes in an Escher picture. The number of rotation axes is the first part of the code to unlock the first lock box, in which a metallographic sample is stored.

*Utilities*: a 25-piece jigsaw puzzle showing an image of a wallpaper group (*e.g.* *p*3*m*1).

Puzzle (2) is a homemade 25-piece jigsaw puzzle showing a graphical representation of a selected wallpaper group. We decided to use *p*3*m*1, *e.g.* found in Escher’s symmetry sketch No. 69 (1948) (https://www.wikiart.org/en/m-c-escher/symmetry-drawing). After the jigsaw puzzle is put together, the rotation axes can be counted. Their number yields the first part of the code.

**Puzzle (3)**

Find the second part of the code, which is written with UV paint on a wall.

**Figure 4:**
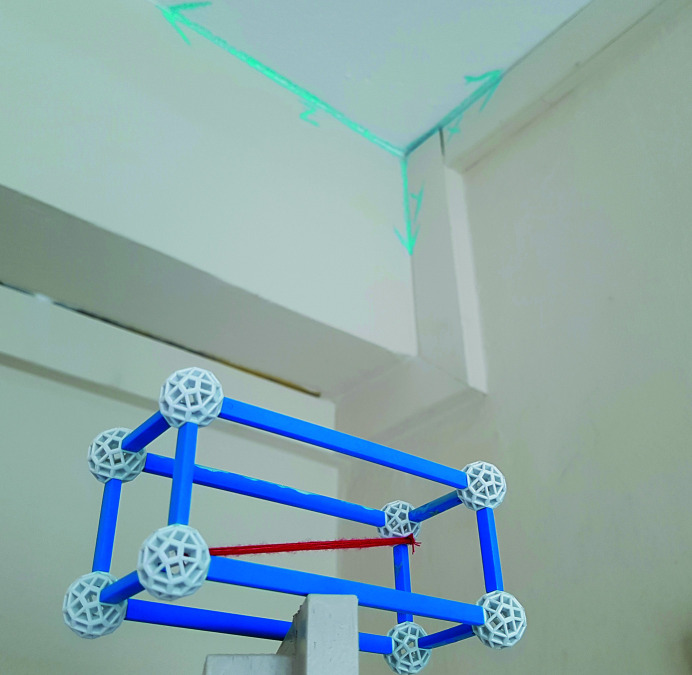
Utility for puzzle (3): orthorhombic unit cell with the [111] direction highlighted.

*Utilities*: a UV lamp and a model of an orthorhombic unit cell with the [111] direction highlighted (Fig. 4 ▸).

*Additional hint*: the second part of the code is (invisibly) located near the ‘endpoint’ of the vector of the [111] crystallographic direction.

The players must transform room 2 mentally into the model of the orthorhombic unit cell provided. A coordinate system is drawn in one corner of the room; this can be seen, together with the unit cell model, in Fig. 4 ▸. When the players illuminate the opposite corner of the room (*i.e.* point 111) with the UV lamp, they will find a formerly invisible number written with UV paint. Combining the numbers from puzzles (2) and (3) reveals the code to open the first lock box. It contains a metallographic sample.

**Puzzle (4)**

Find the error in the elasticity tensor of a hexagonal material. The wrong number is the code for opening the second lock box in room 2.

**Figure 5:**
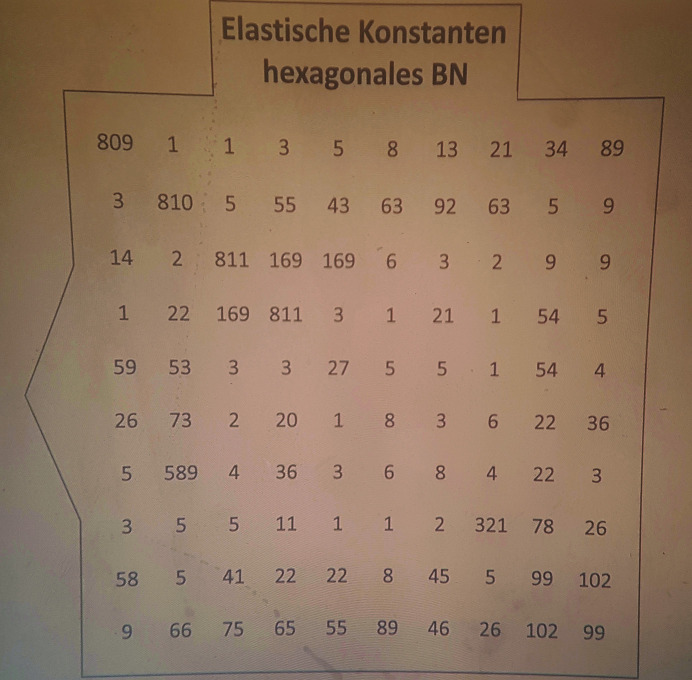
Utility for puzzle (4): a slide with a 10 × 10 matrix containing the elements of the elastic tensor of a hexagonal material.

**Figure 6:**
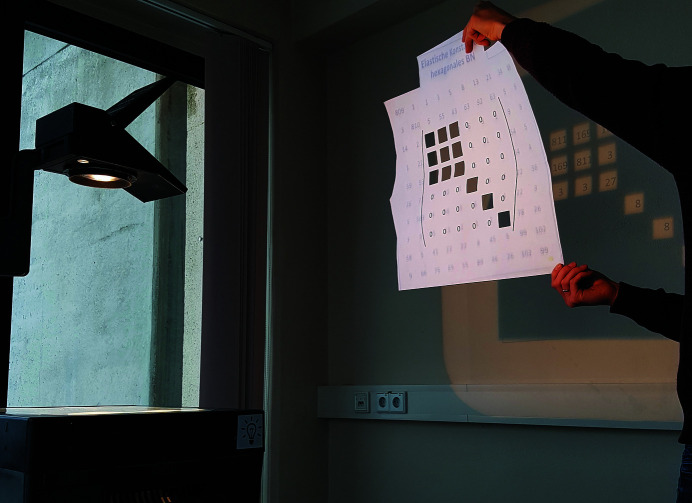
Other utilities for puzzle (4): overhead projector with the slide from Fig. 5 ▸ and a stencil board, which is transparent at the positions of non-zero elements in a 6 × 6 matrix comprising the elastic constants of a hexagonal material.

*Utilities*: an overhead projector, a slide containing a 10 × 10 matrix of numbers (Fig. 5 ▸) and a stencil board covering a 6 × 6 matrix with windows at the positions corresponding to the non-zero elements of the elasticity tensor (Fig. 6 ▸).

*Additional hint*: in Voigt’s notation, the elasticity tensors have the shape of symmetrical 6 × 6 matrices with additional coupling rules for individual elements of the elasticity tensor as imposed by crystal symmetry.

The code needed to open the second lock box in room 2 is the wrong value in the 6 × 6 elasticity tensor. From the whole 10 × 10 matrix, projected on a wall as shown in Fig. 5 ▸, the players must select a symmetrical 6 × 6 matrix that obeys the rules for an elastic tensor of a hexagonal material (in Voigt’s notation). The stencil, which has the form of a 6 × 6 matrix with non-zero elements stamped out (Fig. 6 ▸), helps to identify the part of the 10 × 10 matrix that possesses the required matrix symmetry within the 6 × 6 subset. The symmetry operations in hexagonal materials require the following coupling of the values: *C*_23_ = *C*_13_, *C*_55_ = *C*_44_ and *C*_66_ = (*C*_11_ − *C*_12_)/2.

The single value that violates these additional rules for hexagonal materials is the key to open the lock box. The lock box contains two batteries.

Together with the batteries from puzzle (4) and the metallographic sample from puzzles (2) and (3), the players should pick up a hammer and a chisel, which are stored in the table drawers in room 2. With these items, the players return to room 1 (see Fig. 1 ▸), whose second exit door is locked by a three-digit combination lock. Individual digits are gathered in puzzles (5), (6) and (7).

### 2.3. Back in room 1

**Puzzle (5)**

Retrieve a piece of sand-cast metal to get the first digit of the code, which is needed to open the door to room 3.

*Utilities*: a canister containing moulding sand with a sand-cast piece of metal, a hammer and a chisel.

Using the hammer and chisel from room 2, the players have to retrieve the first digit of the code, which has the form of a sand-cast metal part that is still embedded in moulding sand.

**Puzzle (6)**

Assign the optical micrograph to the sample from room 2 (first lock box) and obtain the second digit of the code for opening the door to room 3.

**Figure 7:**
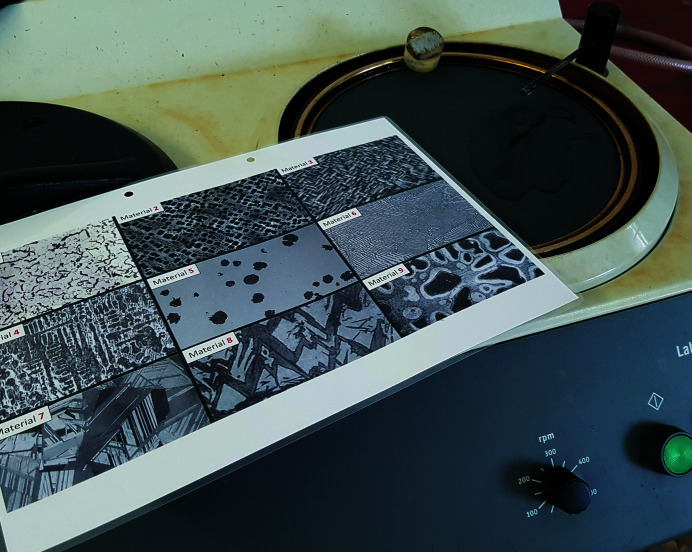
Utilities for puzzle (6): a metallographic preparation unit and optical micrographs with possible microstructure candidates.

*Utilities*: metallographic sample from puzzles (2) and (3), an optical microscope, a metallographic preparation unit and optical micrographs with possible microstructure candidates (Fig. 7 ▸).

*Additional hint*: instructions for the use of the metallographic preparation unit.

As the surface of the metallographic sample is covered by a ‘protective’ layer (*i.e.* painted with an opaque paint), its microstructure is visible using an optical microscope only after this top layer is removed. For this reason, a metallographic preparation unit (Fig. 7 ▸) is employed. After polishing, according to the instructions given as an additional hint, the microstructure of the sample can be visualized by the optical microscope and compared with the optical micrographs provided (Fig. 7 ▸). The number of the matching candidate is the second digit of the code for opening the door to room 3.

**Puzzle (7)**

Determine the distance between the gratings in an optical grid. The distance (in micrometres, rounded to an integer) is the third digit required for opening the door to room 3.

**Figure 8:**
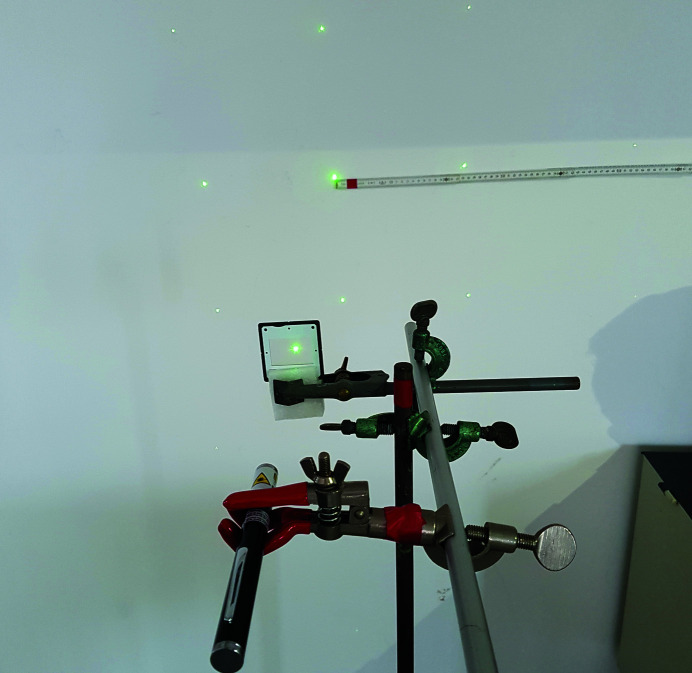
Utilities for puzzle (7): laser pointer, optical grid and a folding yardstick.

*Utilities*: an optical grid, a laser pointer, batteries from puzzle (4), a folding yardstick and a calculator (Fig. 8 ▸).

*Additional hints*: wavelength of the laser light, and a sketch of the experiment with the beam path and the diffraction equation.

The players find a prepared setup of a laser diffraction experiment. A laser pointer is directed towards an optical grid with a bare wall behind. The distance between the gratings in the optical grid is too small to be determined directly using the folding yardstick. Therefore, the diffraction phenomena must be utilized. The laser pointer powered by the batteries from puzzle (4) produces a monochromatic primary beam. The distances between the gratings in the optical grid (*d*) are calculated from the scattering angle using the diffraction equation, *d*sinφ = λ, where φ is the scattering angle, which is the angle between the primary and scattered beams, and λ is the wavelength of the laser light. The scattering angle is calculated from the distance between the grid and the wall on which the diffraction pattern is projected, and from the distance of the diffraction spots from the spot of the primary beam (Fig. 8 ▸). The diffraction of visible light is used as a mock-up for X-ray diffraction and the Bragg equation, which is utilized to determine the distances between lattice planes in crystal structures. Incidentally, the available optical microscope [from puzzle (6)] is inappropriate to resolve the grating distance. The calculated distance (in micrometres) between the gratings rounded to an integer is the third and concurrently the last digit required for opening the door to room 3.

### 2.4. Room 3

Room 3 (see Fig. 1 ▸) is a similar laboratory to room 1. Aside from further puzzles, a briefcase locked with a six-digit code can be found here. This (final) briefcase can be opened using two digits obtained in room 3 and four digits obtained in room 4 (see below). Opening the final briefcase will mark the end of the game. As illustrated in Fig. 1 ▸, room 3 offers two variants of the game.

#### 2.4.1. Room 3: short variant

In the short variant, puzzles (8) and (9) deliver the first two digits to open the briefcase from room 3. The adjacent room 4 is unlocked.

**Puzzle (8)**

Let a number written with transparent hydro­phobic ink on a filter paper appear to obtain the first digit of the code that is needed to open the last briefcase.

**Figure 9:**
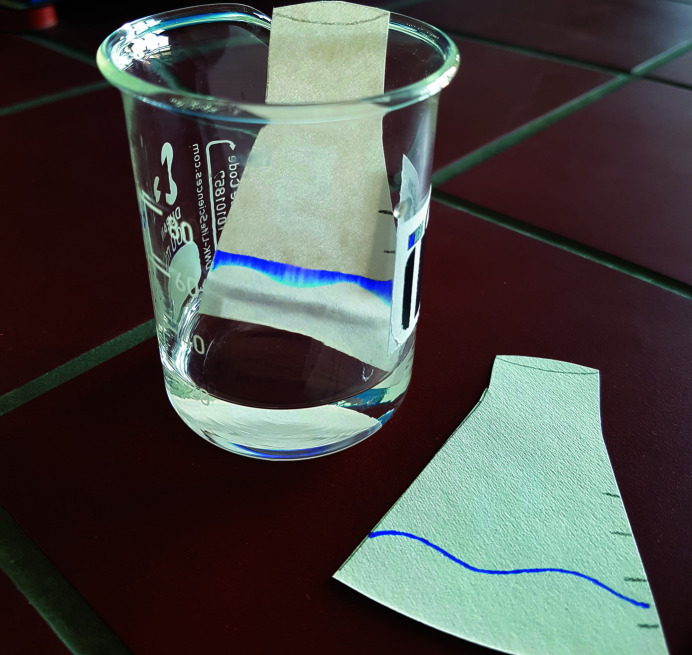
Utilities for puzzle (8): filter paper with a line drawn with coloured hydro­philic ink and with a number drawn with transparent hydro­phobic ink, and a container with water.

*Utilities*: filter paper, transparent hydro­phobic ink, coloured hydro­philic ink and a flask with water (Fig. 9 ▸).

**Figure 10:**
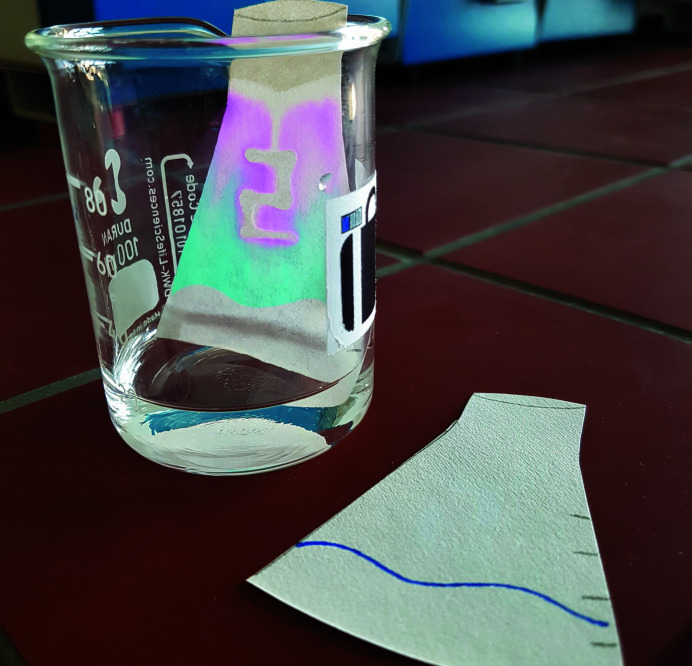
Result of the experiment carried out in puzzle (8). The capillary rise of the water carries the coloured hydro­philic ink towards the top of the filter paper and dyes it. Only the part of the filter paper covered by the transparent hydro­phobic ink remains unaffected. The number sought becomes visible.

This puzzle deals with hydro­philic and hydro­phobic substances. A transparent hydro­phobic ink is used for writing a number on the filter paper. A coloured hydro­philic ink is used to draw a horizontal line on the lower part of the filter paper. Both steps are performed by the ‘game master’ during the preparation of the escape room. The players should immerse the filter paper in the water at a level slightly below the hydro­philic line. The hydro­philic ink colours the water, which rises towards the invisible digit due to the capillary forces (Fig. 9 ▸). The number written by the transparent hydro­phobic ink remains white (Fig. 10 ▸). When coloured water is used, the result is improved.

**Puzzle (9)**

Let a number written on an object hidden in a conical flask filled by hydro­gel balls appear. This number is the second digit of the code that is needed to open the last briefcase.

**Figure 11:**
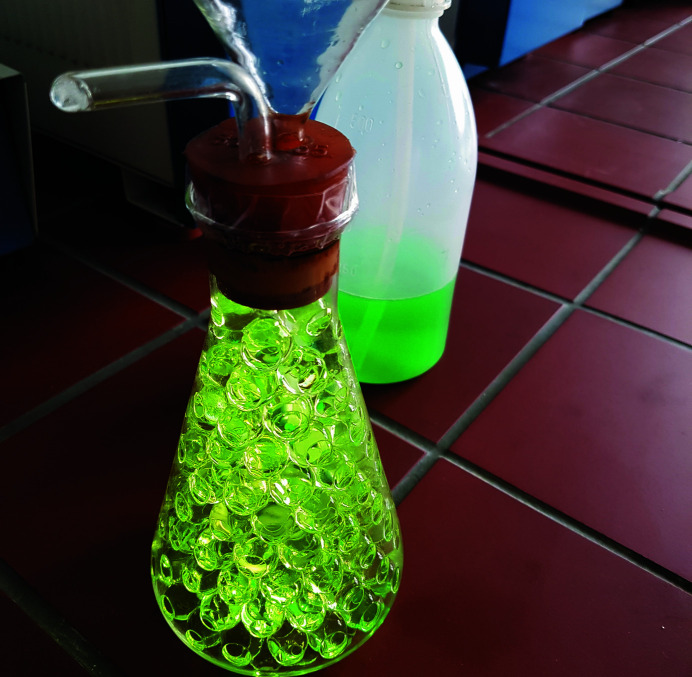
Utilities for puzzle (9): a glass flask filled with hydro­gel balls and a buried object with the sought number.

*Utilities*: a conical flask, hydro­gel balls and a flask with water (Fig. 11 ▸).

**Figure 12:**
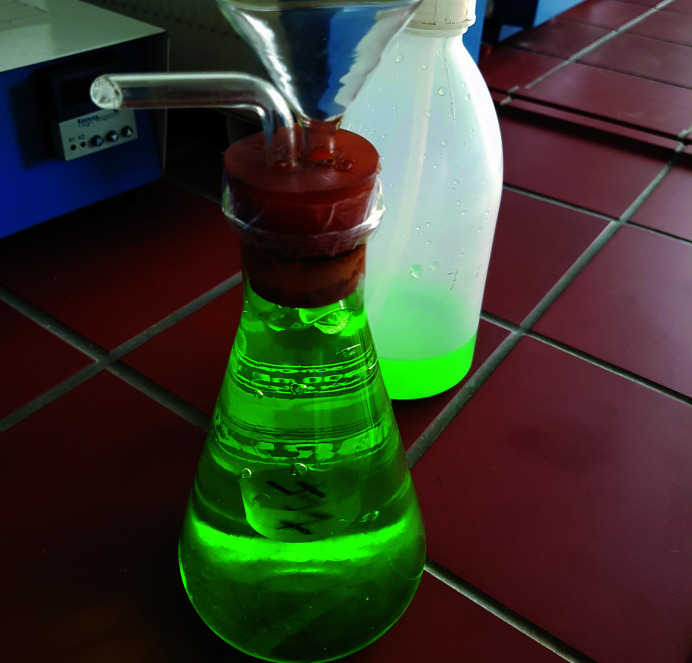
Puzzle (9). The object with the number becomes visible after replacing the air with water.

Puzzle (9) deals with the refraction of light at interfaces between different materials. The players find a conical flask filled with hydro­gel balls (see Fig. 11 ▸) that bury an object, on which a number is written. An additional bottle with water is provided. The object and the number are invisible to the players, because the light entering the flask is refracted at the interfaces between the hydro­gel balls and the surrounding air. Replacing the air with water, which has a similar index of refraction to the hydro­gel balls, eliminates the refraction at the interfaces and makes the buried object with the number visible (see Fig. 12 ▸). The refraction indices of water and the hydro­gel balls are similar because the hydro­gel contains more than 95% water. Thus, light entering the flask can pass through it almost without being refracted when the flask is filled with water instead of air. The number seen on the object in the conical flask is the second digit required to open the briefcase. Using coloured water results in a more impressive view.

#### 2.4.2. Room 3: long variant

In the long variant, a three-digit code to open a lock box found in room 3 containing the key to room 4 must be gathered from puzzles (10), (11) and (12). Puzzle (13) yields the first two digits for the final briefcase, which are obtained from puzzles (8) and (9) in the short variant.^1^

**Puzzle (10)**

Identify the metal with the lowest melting point and melt it using an infrared lamp. In the metal, a number is buried, which is the first digit of the code to open the lock box in room 3.

**Figure 13:**
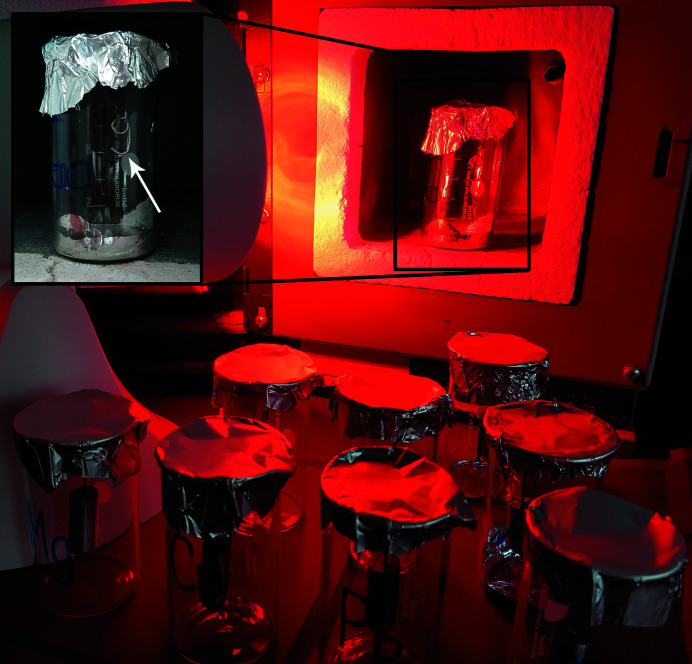
Utilities for puzzle (10): flasks with metal ingots, an infrared lamp, fireproof environment (oven).

*Utilities*: ten transparent flasks each containing a different piece of an elemental metal and an infrared lamp (Fig. 13 ▸).

*Additional hints*: each flask is labelled by the chemical symbol of the metal, a table displays the melting points of the metals provided.

Among all the metals provided, the players should identify the metal which melts at a temperature that can be generated by the infrared lamp. Small ingots of ten metals hang in the flasks. The flasks are labelled with the chemical symbol of the metal inside. The metal with the lowest melting point (Ga) conceals the first digit of the three-digit code to open the lock box. As a hint, a table with the melting points of all metals provided is given. After the correct selection, the players must melt the low-melting metal with the infrared lamp (Fig. 13 ▸). After the hanging metal ingot is melted (and the metal drops down), it reveals a number, which is the first digit of the code needed (insert in Fig. 13 ▸). The game master must be present during this experiment, due to the possible hazards associated with Ga. The players must not touch the Ga with bare hands.

**Puzzle (11)**

Identify a metal, from which a cylindrical sample is made, with the aid of its characteristic X-ray spectrum obtained by energy-dispersive X-ray spectroscopy (EDX). Determine its density (g cm^−3^; rounded to the nearest integer), which is the second digit of the code to open the lock box in room 3.

**Figure 14:**
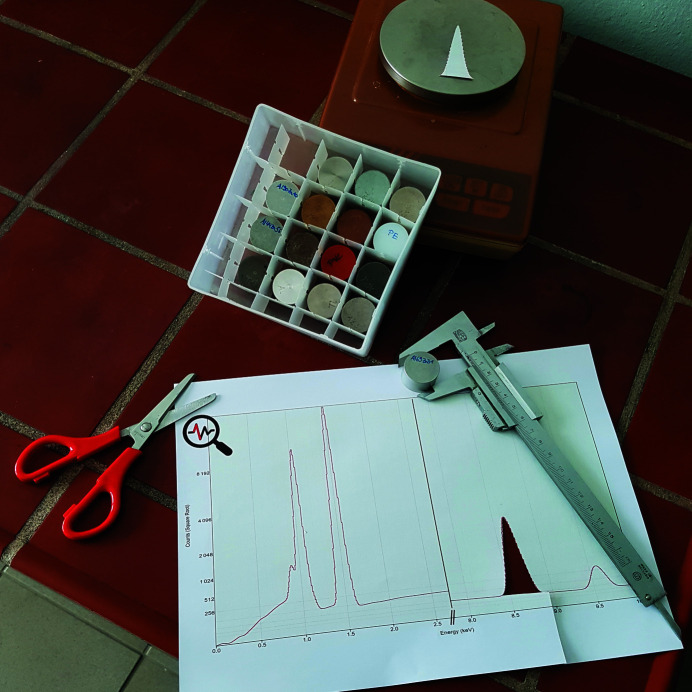
Utilities for puzzles (11) and (12): set of materials with different densities, balance, calliper, part of the characteristic X-ray spectrum, scissors.

*Utilities*: a printout of the EDX spectrum, labelled cylinders made of different metals, a scale, a calliper, a calculator (Fig. 14 ▸) and a table listing the energies of the emission lines of the elements.

*Additional hints*: explanation of the ionization process and the creation of characteristic emission lines.

The players are provided with an EDX spectrum of an unknown substance and with a box containing labelled cylinders made from different materials. The EDX spectrum shows selected X-ray spectral lines, which are typical for the material sought. The players must compare the peak energies from the spectrum with the peak energies given in the table with the X-ray emission lines. In this way, the elements in the sample can be identified. On the basis of the labels written on the cylinders, the players can now select the correct cylinder from the candidates placed in a box. Using the scale to weigh the sample and the calliper to determine the cylinder volume, the density of the correctly identified material can be calculated. The density (g cm^−3^; rounded to the nearest integer) is the second digit of the lock box code. The materials used for puzzle (11) and (12) are shown in Fig. 14 ▸.

**Puzzle (12)**

Identify a metal on the basis of its EDX spectrum. Its density (in g cm^−3^, rounded to the nearest integer) is the third digit of the code to open the lock box in room 3.

*Utilities*: a printout of the EDX spectrum on cardboard, a table with emission line energies of the elements, labelled cylinders made of different metals, a scale, a calliper, a pair of scissors and a calculator (Fig. 14 ▸).

*Additional hints*: explanation of the information content of the peak area.

Puzzle (12) involves the same setup as puzzle (11) but with a different EDX spectrum. Thus, players again must identify the elements in the spectrum to find the correct cylinder. The difference from puzzle (11) is that three cylinders from the box are identified as possible candidates according to the energies of their emission lines. These samples contain the same two elements but in different amounts. This information is hidden in the integral intensities of the strongest EDX lines (areas below the strongest EDX peaks), which can be determined by weighing the peaks cut out of the printed spectrum. The ratio of the integral intensities (weights) of the strongest peaks stemming from two individual elements, which corresponds to the ratio of the elements, identifies the right candidate. When the cylinder is correctly identified, its density (in g cm^−3^, rounded to the nearest integer) must be determined in the same way as in puzzle (11) to obtain the third digit of the lock box. For this puzzle, cylinders made from Al and Zn were used because these samples are easy to manufacture and show a clear change in density as a function of their chemical composition.

Together, puzzles (10), (11) and (12) yield the code to the lock box found in room 3, which contains the key to room 4. Room 3 also contains puzzle (13), which delivers the first two digits of the final six-digit code to open the briefcase marking the end of the game.

**Puzzle (13)**

Find a stellar constellation among many candidates depicted in the table. The numbers of the corresponding rows and columns in the table are the first two digits of the final six-digit code to open the briefcase.

**Figure 15:**
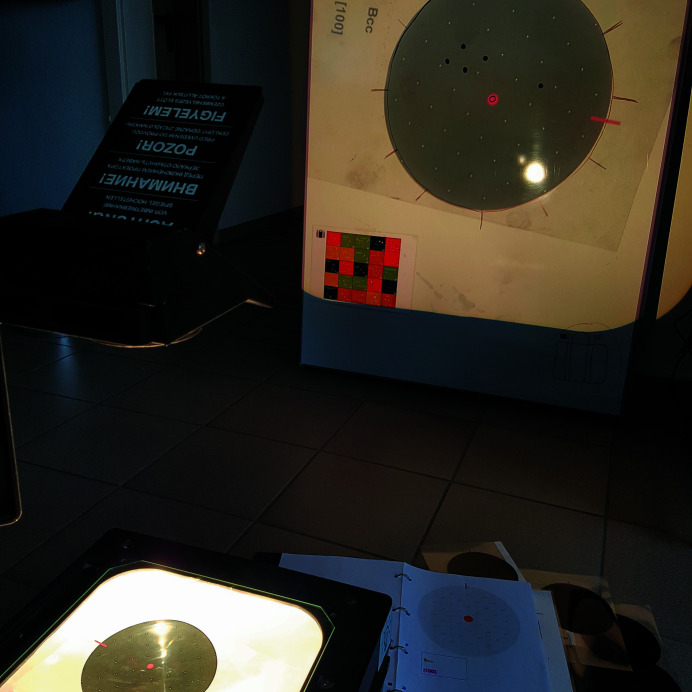
Utilities for puzzle (13): overhead projector, single-crystal diffraction pattern on a transparency, polar diagram on a whiteboard, table (checkerboard) with stellar constellations.

*Utilities*: single-crystal diffraction patterns on transparencies, an overhead projector, a polar diagram on a whiteboard, a whiteboard pen (Fig. 15 ▸) and a table with stellar constellations.

*Additional hints*: a list containing the diffraction patterns to be used, the respective diffraction spot of interest in each pattern and the rotation to be applied to the diffraction patterns.

In this puzzle, the players are confronted with the rotation of the diffraction patterns from single crystals which occurs during the rotation of the sample. The setup is shown in Fig. 15 ▸ and the result of the puzzle is a set of points appearing on the whiteboard. The hint given for this puzzle is a list that motivates the players to select a requested diffraction pattern and put it on the overhead projector to project it onto the whiteboard. Then, a certain diffraction spot as given in the list must be selected and the pattern must be rotated around its centre about an angle also given in the list. The final position of the selected diffraction spot must be marked on the whiteboard. Performing this operation with all diffraction patterns given in the list produces an arrangement of dots on the whiteboard that assumes the shape of a stellar constellation. From a table with different stellar constellations (the checkerboard in Fig. 15 ▸) the correct one must be identified. The row and line number of the correct solution in the table give the first two digits of the final six-digit code.

### 2.5. Room 4

The final room can be accessed either directly from room 3 (short variant) or by unlocking it using the key found in the lock box opened with the digits obtained in puzzles (10), (11) and (12) in room 3 (long variant). The room resembles an office with a computer and a telephone, where publications are written or meetings with colleagues are held.

**Puzzle (14)**

Obtain the remaining four digits for the briefcase by calling a colleague.

*Utilities*: a computer with a 3.5′′ disc drive, the 3.5′′ floppy disc found in the lock box in room 1 which has been opened after solving puzzle (1) and a prepaid mobile phone.

Young people must recognize that the 3.5′′ floppy disc, which they have found in the lock box that opened after puzzle (1) was solved, is an old electronic storage medium. Players immediately recognize the progress that has been made in the field of data storage since the 1980s. Inserting the 3.5′′ floppy disc into its drive provides access to a single text file on this disc. The text file contains a telephone number. On using the telephone provided and calling the number, an electronic voice can be heard saying the remaining four digits of the six-digit code of the briefcase. Combining these four digits with the two digits obtained in puzzles (8) and (9) (short variant) or puzzle (13) (long variant) gives access to the content of the briefcase and marks the end of the game.

In the briefcase, the players will find a participation certificate and some giveaways. On the back of the participation certificate there is a summary of the physical background of all the puzzles that links them to materials science and crystallography. An example of the participation certificate can be found in the supplementary material. In a debriefing with the players after the game, the game master discusses the puzzles and their relation to materials science in detail. Importantly, the players can also talk about their experiences during gameplay. During this debriefing, the story is closed. The game master points out that conferences and international cooperation are another important aspect in the job of a scientist. It is argued that the abandoned laboratory with the unfinished experiments results from the urgent travel of the scientists to an important international conference.

## 3. Subjective observations regarding the players’ habits and implications for an escape room setup

The escape room described above was established in 2019. It was tested in several runs performed with the academic staff of the Institute of Materials Science. Subsequently, it was opened for the public. The players are mainly pupils from secondary schools, but teachers and undergraduate students have also been welcomed. Pupils visit the escape room usually in the frame of trips to the university campus, which are organized by their schools. The general feedback of the players is overwhelmingly positive. The players are often fascinated by the appearance of known physical effects in a context they did not expect or the observation of phenomena that were previously unknown to them. Teachers usually appreciate that mathematical principles or physical effects are illustrated in a practical context. In particular, the final debriefing helps the players to understand the scientific background of individual puzzles. Very often, some puzzles are repeated together with an explanation of the underlying principles and phenomena by the game master. We found that this is a very important measure to stimulate the players’ interest in physical or chemical phenomena. We also found that it was very helpful to let the players talk about their experience, not only for their own understanding of the background of the puzzles but also as feedback for the game master to improve the escape room setup.

Regarding the game play, we found significant differences in player performance. The quickest groups are those whose players are motivated, and which are characterized by a high degree of internal communication and teamwork, as found by Veldkamp *et al.* (2020 ▸, 2021 ▸). Groups from a school with a dedicated STEM profile performed with more ease than groups from other schools on average. Good performances are also delivered by groups taking part in the escape room completely voluntarily. Furthermore, groups with a ‘leader’ are faster than groups without a hierarchical system. This hierarchy evolves often during play. It is facilitated by the dynamics of the group (*i.e.* not imposed by the game master). As some of the tasks/puzzles can be solved in parallel, groups that are able to divide quickly into sub-groups deliver the best performance combined with the lowest intervention of the game master.

On the other hand, parallel tasks that are not solved with approximately the same speed can motivate the players to go for a trial-and-error method to open a case when they know two digits of a three-digit code. This motivation may be stronger when the players’ frustration increases or when a sub-group waiting for another sub-group is bored. Sometimes, the time to solution is longer, because especially younger players are anxious that they may do something wrong or that they may destroy the equipment. This habit quickly decreases after the first one or two puzzles have been solved. When there are frustrations resulting from the trials to open a case by searching for a correct last digit, the game master should motivate the players to solve the puzzles in the intended way, *e.g.* by supporting the delayed players in their search for the solution (*i.e.* partially disclosing the approach to solve the puzzle) or by involving the members of other sub-groups in the solution. To avoid the boredom situation *a priori*, the game master should try to ensure that the sub-groups proceed at approximately the same speed so that all numbers of the codes that are still required become available simultaneously. A general experience is that the higher the players’ motivation, the less frequently players use trial-and-error approaches to open the case.

An objective assessment of the achievements of the players in an escape room is not easy (Lathwesen & Belova, 2021 ▸). Still, after welcoming more than 50 groups in our escape room, we can conclude that, irrespective of age and previous knowledge of the players, all puzzles *can* be solved without interaction with the game master. To guide the players towards the puzzle solution, most of the puzzles are accompanied by a number of hints (‘how to’ notes; see supporting information). This is especially helpful when the puzzle contains scientific content that is new to the players. For content known to the players, no hints are required. In this context, we recognize that the motivation to read the hints decreases during the playtime. Thus, if hints are provided, they should contain as little text as possible. Sketches or comics have been found to be valuable in that context. If the concentration of the players declines, additional hints from the game master may be useful. Often players possess the knowledge to solve the puzzles but do not recognize the context in which they should apply it. Furthermore, the presence or absence of the hints can be used to create different levels of difficulty of the escape game and make it challenging for player groups of different levels of knowledge. Generally, motivated and enthusiastic groups are able to solve the escape room in the expected time (or even less). The intervention of the game master can speed up the game process if necessary. However, care must be taken in order not to spoil the experience of autonomy, discovery and victory for the players during the gameplay (Veldkamp *et al.*, 2021 ▸).

## 4. Summary

We transformed parts of a laboratory facility and an adjacent seminar room – which were initially designed for university laboratory courses – into an escape room. The escape room can be set up within an hour by installing incomplete experiments (*i.e.* puzzles) that need to be completed by the players in order to finish the game. The initial laboratory layout can be easily restored, allowing us to use limited laboratory space for both regular academic teaching and hosting this escape room. The authentic atmosphere of the rooms offers a holistic experience through puzzles that address different areas of materials science (like solid-state physics, crystallography, metallography, chemistry, microscopy, and X-ray and electron diffraction). The certificate with the explanations of the experienced phenomena on the back help to keep the event and the acquired knowledge memorable for a long time. A debriefing helps the players to reflect on their game play experiences and comprehend the scientific background in the puzzles.

Positive feedback from players and accompanying teachers (in the case of pupils) has been received for the authenticity of the setting and the bandwidth of puzzles touching many scientific disciplines. Using scientific phenomena (*e.g.* breakdown of ferromagnetism, diffraction of visible light) to create a (sometimes unexpected) effect to solve a puzzle is a high motivation for the players to engage themselves in the science. Also, pupils are happy when they are able to apply some knowledge from school to solve a problem. Furthermore, the players often appreciate that they can split into groups and that results from different groups are combined to open a combination lock. This parallelism of puzzles is sometimes also perceived in a negative sense because not all players can have the same experience. A repetition of the puzzle solution during debriefing usually resolves this issue. Teachers enjoy observing the interactions between their pupils in a way that is almost impossible during normal school classes, as the players are, for example, free in the interactions with the other players and in their selection of puzzles they want to solve. After finishing the escape game, players typically confess that the provided hints would have been helpful during gameplay but also that they were often too excited to read them carefully or to comprehend their meaning. It also takes some warm-up time until the players have adjusted themselves to the inherent logic of the puzzles and the escape game in general. During that time, the degree of intervention of the game master is critical, between assistance in the beginning of the game on one hand and damage to the authenticity of the setting or demotivation of the players on the other hand.

In conclusion, this escape game appears as a good opportunity to sensitize pupils and teachers to the importance of natural sciences in general and materials science in particular in view of the current societal challenges. By varying the duration of the game, and the number and the depth of the hints, this concept is suited to offering a challenging escape room experience to a wide range of players (pupils, students and scientific staff) as it creates a flexible level of complexity, variable game play duration and a variety of different puzzle topics. We hope that we can excite young people about STEM in general and materials science and crystallography in particular by using our escape room. This effort is further consolidated when the accompanying teachers include aspects of materials science or crystallography in their courses.

## Supplementary Material

Example hints for the escape room used to adjust the level of difficulty. DOI: 10.1107/S1600576723006714/dv5005sup1.pdf

## Figures and Tables

**Table 1:** Scientific backgrounds of the puzzles described in the text and a compilation of the materials used to create them

Puzzle	What should be learned/scientific background	Materials and setup	Cost (if >10 euro)
(1)	Electrical current produces Joule heat; the Curie temperature marks the upper temperature limit of ferromagnetism	Ni wire (∅ 0.5 mm), electrically insulating clamps, power supply unit (∼10 V, ∼10 A), screws, scale, Fe–Nd–B magnet, hint with average screw weight	∼10 euro for 25 m Ni wire (single), ∼10 euro for screws (single), transformer is university equipment
(2)	The structure of crystalline solids is described by periodically arranged units (unit cells); crystal structures possess symmetry	Large wooden board, large printout of image, saw to make a jigsaw puzzle	∼10 euro for wooden board (single)
(3)	Directions in crystals can be described using simple vector algebra known from school mathematics	Zometool (or similar) to build a unit cell, UV pen and UV torchlight, marked coordinate system in room	∼10 euro for UV torchlight (single)
(4)	Elastic constants of crystals are anisotropic (dependent on the crystallographic direction) but obey symmetry rules	Overhead projector, transparency with matrix, large printout (120 g m^−2^ paper) of stencil with holes at correct positions	Overhead projector is university equipment
(5)	Moulding sand is used to give cast objects their shape	Tin can, moulding sand, piece of metal in shape of a number, oven (approximately 100°C), chisel, hammer	Moulding sand available through the institute
(6)	Solids have an internal microstructure (as much as biological cells do); grinding and polishing (and etching) can be used to make the microstructure visible by observation with an optical microscope	Grinding paper FEPA P 2400 and 4000 (metallography supply), metallographic polishing equipment (alternatively, a belt sander or even manual grinding and polishing), water supply, embedded sample silver ink pen, optical reflection microscope, images of candidate microstructures	∼5 euro for each grinding paper (must be replaced from time to time), optical microscope is university equipment
(7)	Monochromatic light is diffracted at translationally periodic objects, when their separation distance is of the same order of magnitude as the wavelength of the light; diffraction can be used to measure distances between lattice planes in crystals	Laser pointer, grating (from optics supply or a fine sieve with equidistant mesh), folding yardstick, laptop or scientific calculator	∼30 euro for laser pointer (single), ∼10 euro for grating foil (single), laptop
(8)	Substances can be hydro­philic or hydro­phobic	Water, filter paper (*e.g.* coffee filter), prepared in nice shape and with a hydro­philic ink mark or line as well as a white/transparent hydro­phobic number [using *e.g.* the UV pen from (3)]	
(9)	Light is refracted at interfaces of materials only when they have different refraction indices	Conical flask, hydro­gel balls, water, object with number	∼10 euro for 500 hydro­gel balls (might require replacement from time to time)
(10)	Light bulbs produce heat, which can melt low-melting metals; at the melting point metals become liquid; melting points of metals can differ by several orders of magnitude	Red light lamp, small oven or insulated box, piece of Ga, reusable mould (*e.g.* test tube or sample vial), wire folded as number to freeze in Ga, several machined metal pieces in the same shape, flask cover (*e.g.* Al foil), table with melting points of the metals	∼30 euro for red light lamp (single), ∼400 euro/100 g Ga (single), other metal pieces were in average ∼10 euro each
(11)	Each chemical element has specific, characteristic X-ray emission lines, which can be used for their identification	Printout of simulated or measured EDX pattern, reference energy table, selection of identical cylinders made of different materials, scale, calculator, calliper	Cylinders were made in the institute workshop, on average 10 euro per cylinder
(12)	Intensities of the characteristic X-ray emission lines are used for the quantification of elements in compounds	Same as (11) but printout on cardboard, scissors	
(13)	Rotation of a single crystal in a diffraction experiment results in rotation of its diffraction pattern	Printouts of simulated electron diffraction patterns on transparencies, overhead projector, whiteboard with pens, list of stellar constellations, manual with instructions	Overhead projector is university equipment
(14)	Experiencing scientific and technical progress over time	3.5′′ floppy disc, prepaid mobile phone, computer with floppy disc drive	∼10 euros for mobile phone SIM card (single), prepaid credits must be recharged, second hand mobile phone

			**Total: ∼700 euro (with ∼50% of it for the Ga metal)**

## Footnotes

1 Puzzles (8) and (9) from the short variant and puzzle (13) from the long variant deliver the same numbers so that the code of the briefcase does not necessarily need to be changed between the two variants.
