# Supplementary material for: Escape our Lab: creating an escape room game in the field of materials science and crystallography
Source: J Appl Crystallogr. 2023 Aug 18;56(Pt 5):1544–56. doi: 10.1107/S1600576723006714 (PMC10543670; doi:10.1107/S1600576723006714)
Supplement: Supplementary file 1 [file j-56-01544-sup1.pdf]

JOURNAL OF APPLIED CRYSTALLOGRAPHY

Volume 56 (2023)

Supporting information for article:

Escape our Lab – creating an escape room game in the field of materials science and crystallography

Christian Schimpf, Jonas Lachmann, Marius H. Wetzel, Peter D. B. Fischer, Andreas Leineweber and David Rafaja

In the following, we present some images of our participation certificates and the design of our hints as stimulation for readers who want to develop our ideas further.

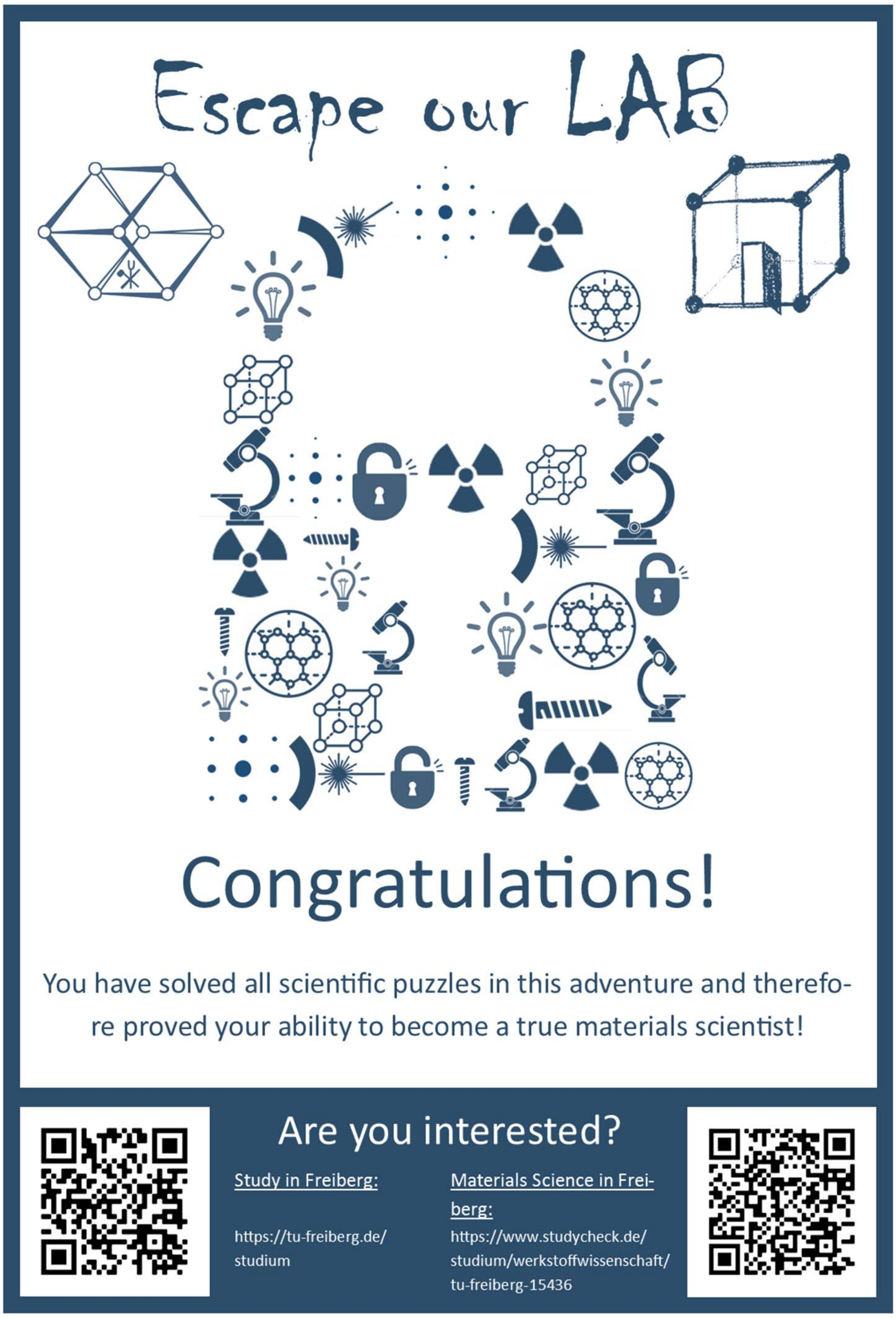

Figure S1 Participation certificate including links to web domains related to studies at our university and to an independent portal with information on the course ‘Materials Science and Engineering’ at the TU Bergakademie Freiberg.

Puzzle	Puzzle core	Application/knowledge
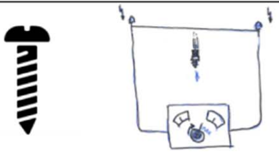	• Curie temperature	• Ferromagnetism breaks down above the Curie temperature.<br>• A metallic wire heats up when an electric current passes through it (Joule heating).
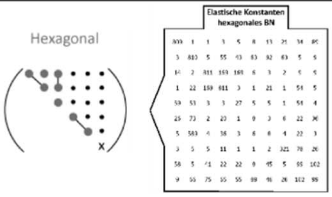	• Tensor of the 2nd order elastic constants	• Elastic properties of crystalline matter depend on the crystallographic direction (anisotropy).<br>• Anisotropic material constants are formulated in a tensor, covering the 3D properties.
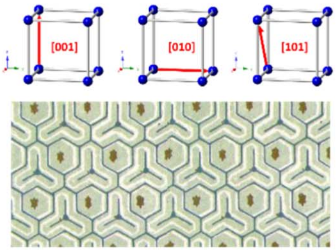	• Unit cell<br>• Miller indices<br>• Symmetry	• Crystalline lattices are composed of repeating units: the unit cell.<br>• Directions and planes inside the unit cell are given by 3-digit numbers (vectors for directions, Miller indices for planes).<br>• Crystalline lattices obey highly symmetric construction principles (involving e.g. rotation axes, mirror planes, etc.).
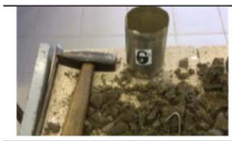	• Casting technology	• Sand moulds are used to cast liquid metals into specific shapes.<br>• After solidification, the sand must be removed to recover the metal piece.
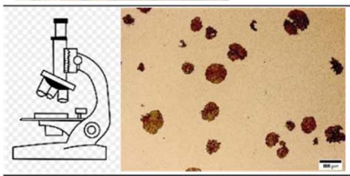	• Sample preparation<br>• Optical microscopy	• Optical microscopes reveal the internal structure (microstructure) of a material.<br>• Before observation, the sample must be ground and polished to generate a mirror-like surface free of scratches
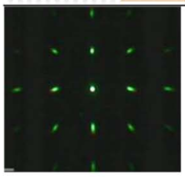	• Optical diffraction	• Electromagnetic waves are diffracted at periodic lattices (gratings) leading to interference patterns.<br>• The interference pattern can be used to estimate the distance between the scattering centres (e.g. lattice planes).
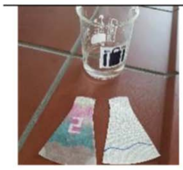	• Hydrophilic and hydrophobic substances	• Polar (hydrophilic) molecules are well miscible with polar water molecules.<br>• Waterproof (hydrophobic) pen colour does not smudge when getting in contact with water.
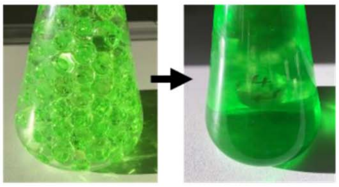	• Optical diffraction at interfaces	• Light changes its direction at interfaces of media having different index of refraction.<br>• Soaked hydrogel balls have virtually the same index of refraction as water, thus eliminating refraction when water is immersed between the balls.
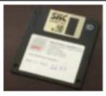	• 3.5” floppy disc	• A storage medium nowadays being supported only by a few electronic systems.

Figure S2 Take-home messages for the short variant of the game, including short descriptions of the scientific background of the game.

Puzzle	Puzzle core	Application/knowledge
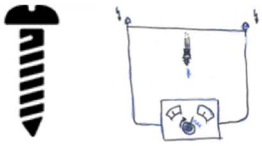	• Curie temperature	• Ferromagnetism breaks down above the Curie temperature.<br>• A metallic wire heats up when an electric current passes through it (Joule heating).
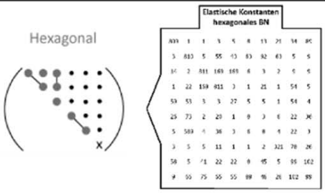	• Tensor of the 2nd order elastic constants	• Elastic properties of crystalline matter depend on the crystallographic direction (anisotropy).<br>• Anisotropic material constants are formulated in a tensor, covering the 3D properties.
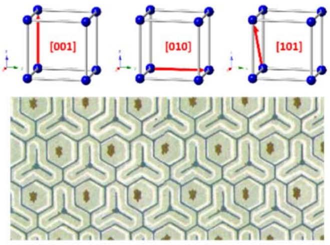	• Unit cell<br>• Miller indices<br>• Symmetry	• Crystalline lattices are composed of repeating units: the unit cell.<br>• Directions and planes inside the unit cell are given by 3-digit numbers (vectors for directions, Miller indices for planes)<br>• Crystalline lattices obey highly symmetric construction principles (involving e.g. rotation axes, mirror planes, etc.).
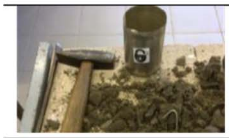	• Casting technology	• Sand moulds are used to cast liquid metals into specific shapes<br>• After solidification, the sand must be removed to recover the metal piece.
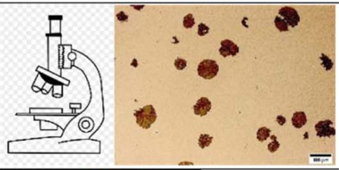	• Sample preparation<br>• Optical microscopy	• Optical microscopes reveal the internal structure (microstructure) of a material.<br>• Before observation, the sample must be ground and polished to generate a mirror-like surface free of scratches.
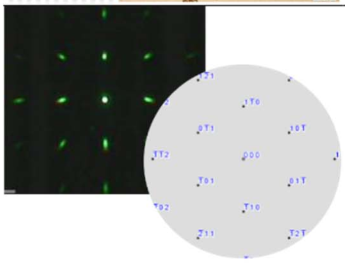	• Optical diffraction<br>• Electron diffraction	• Electromagnetic waves are diffracted at periodic lattices (gratings) leading to interference patterns.<br>• The interference pattern can be used to estimate the distance between the scatterers (lattice planes)<br>• in single crystals, zone axis and symmetry of the lattice determine the interference pattern
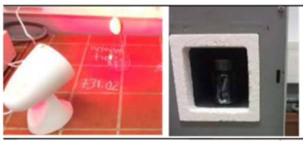	• Melting of Gallium	• Gallium melts at ~30°C, slightly above room temperature<br>• a conventional red light lamp can be used to melt this metal
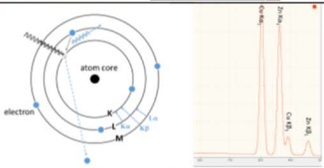	• EDX (energy dispersive X-ray spectroscopy)	• method to determine the chemical composition of a material<br>• EDX spectra are used to identify and quantify the chemical elements contained in (technological) materials
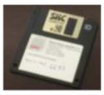	• 3.5" floppy disc	• A storage medium nowadays being supported only by a few electronic systems.

Figure S3 Take-home messages for the long variant of the game, including short descriptions of the scientific background of the game.

To-do list for lab assistants

Task	Done
Prepare lab for pupils	✓
Count screws	
Clear tables in the seminar room	
Remove cast metal from the sand form	
Count rotation axes in jigsaw puzzle; what is at the end of the highlighted direction	
Identify error in the elastic constants of hexagonal BN	
Check the lattice constant of the grating for the practical course ‘laser diffraction‘ (rounded in µm)	
Prepare sample C47b and identify the material	
Fill the Erlenmeyer flasks	
Call the head of the lab	

Figure S4 To-do list as ‘guidance’ through the whole escape room (short version).

To-do list for lab assistants

Task	Done
Prepare lab for pupils	✓
Count screws	
Clear tables in the seminar room	
Remove cast metal from the sand form	
Count rotation axes in jigsaw puzzle; what is at the end of the highlighted direction.	
Identify the error in the elastic constants of hexagonal BN	
Check the lattice constant of the grating for the practical course ‘laser diffraction‘ (rounded in µm)	
Prepare sample C47b and identify the material	
Melt metal	
Identify samples of which the EDX spectra were taken	
Determine their density in g/cm^3 (round to integer)	
Assign electron diffraction patterns to the correct image	
Call the head of the lab	

Figure S5 To-do list as ‘guidance’ through the whole escape room (long version).

Below, we show our additional hints that may be provided to the players depending on their prior education or knowledge. For secondary school pupils, we found that most of them are relevant. For undergraduate students, almost none of them needs to be provided. For other player groups, notes with a reduced information depth may be used/prepared. The original versions of the additional hints are handwritten. This gives them a higher authenticity as laboratory notes.

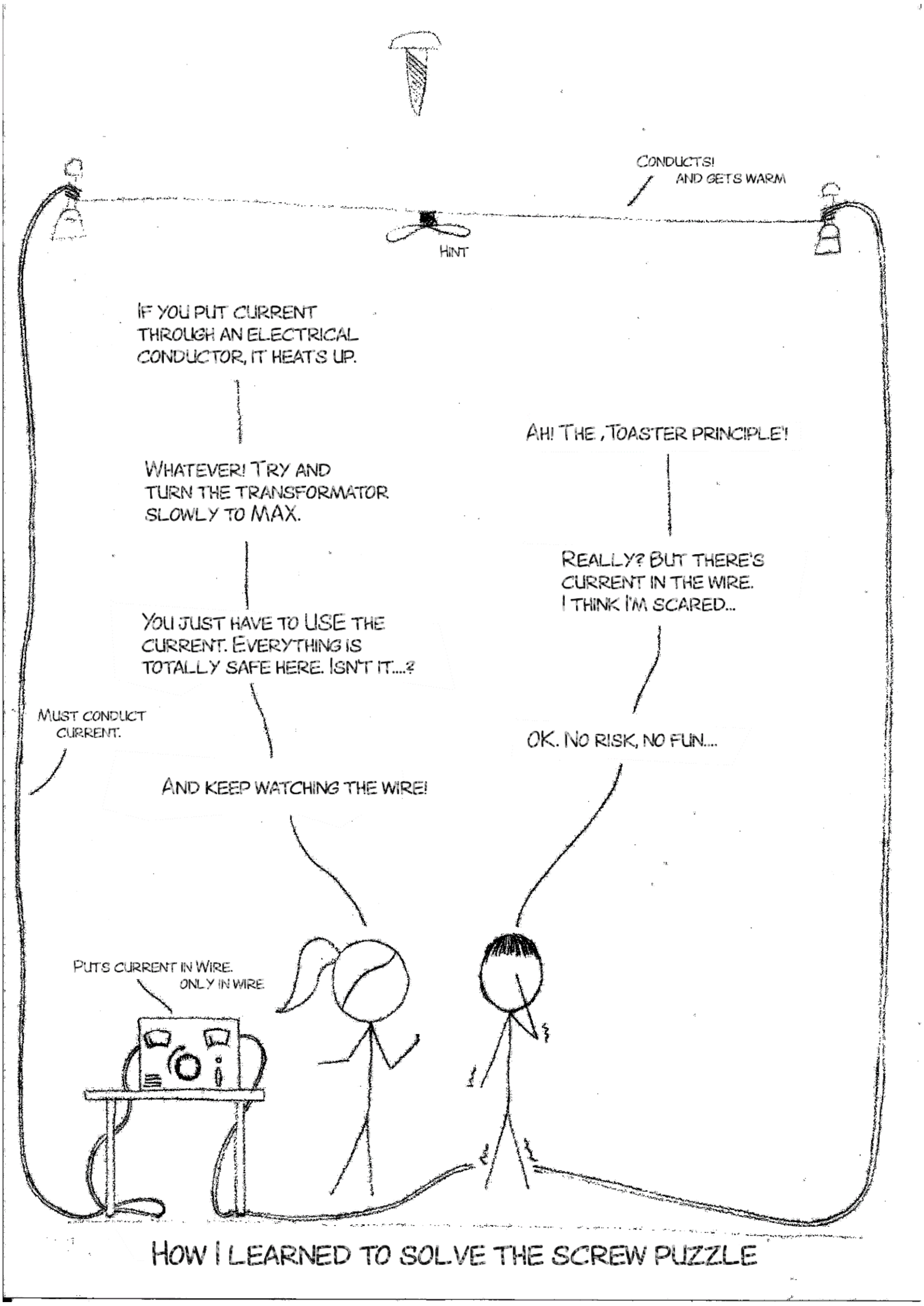

Figure S6 Additional hint for puzzle (1).

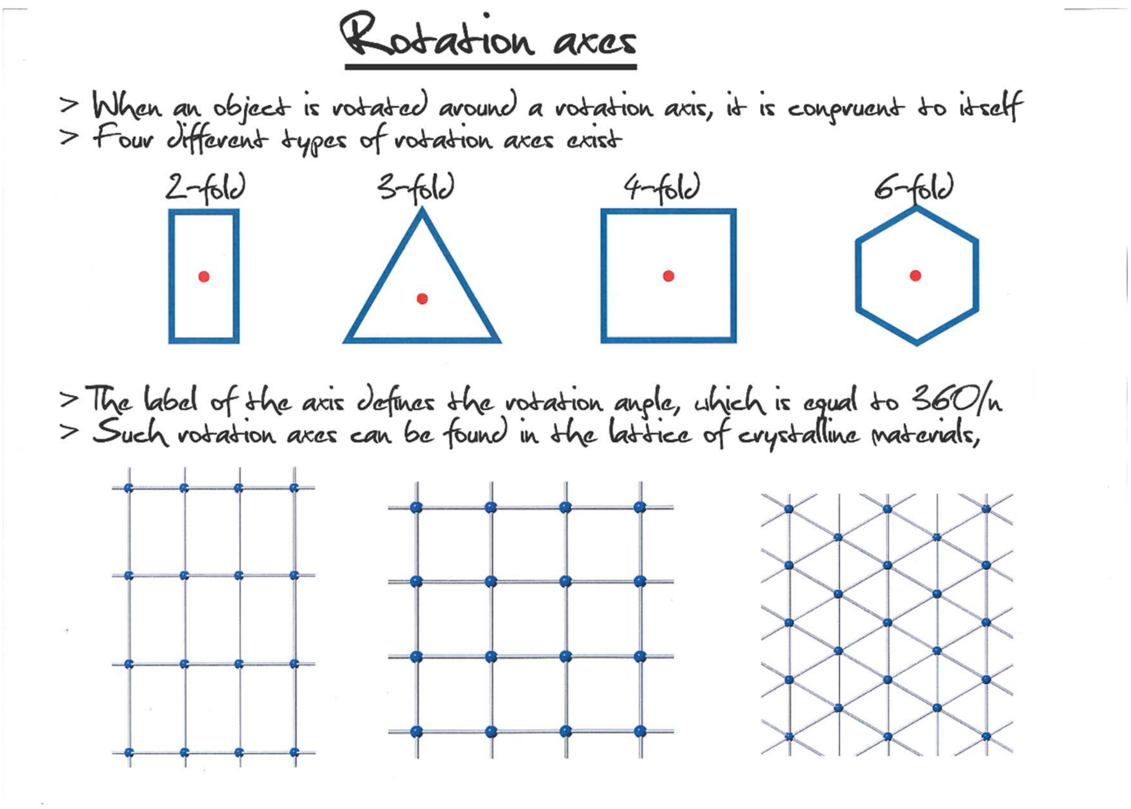

Figure S7 Additional note for puzzle (2).

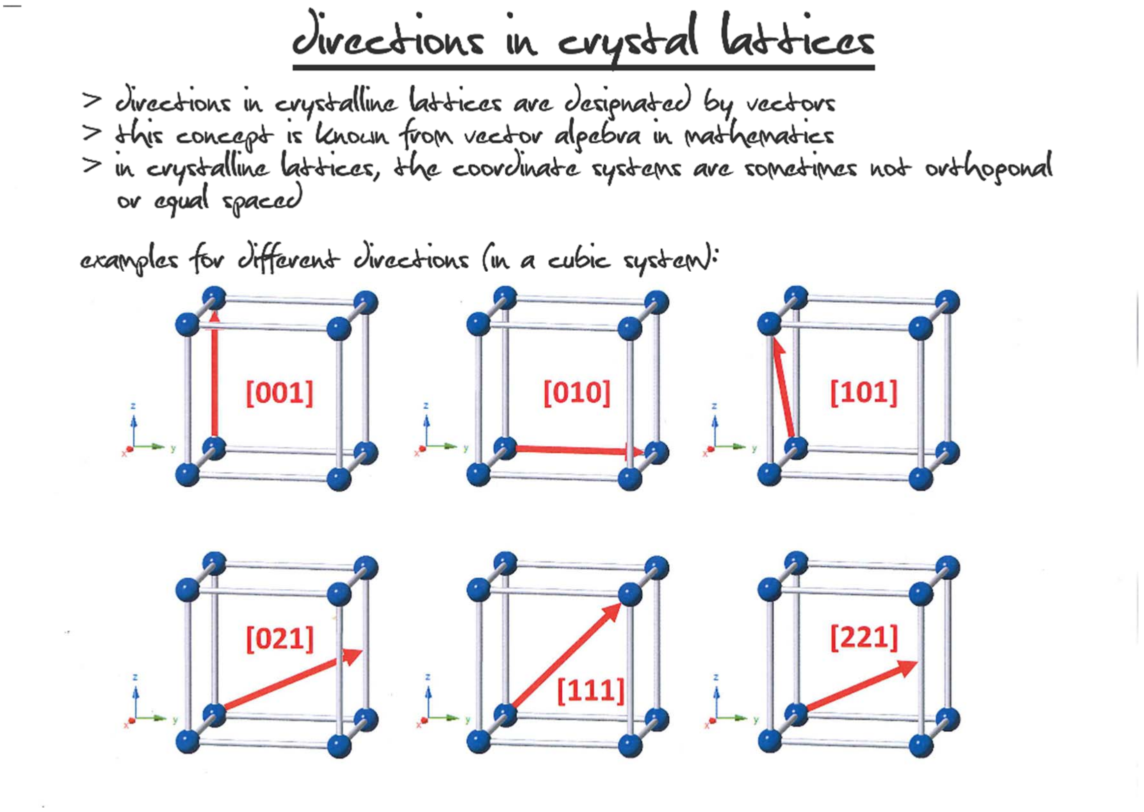

Figure S8 Additional note for puzzle (3).

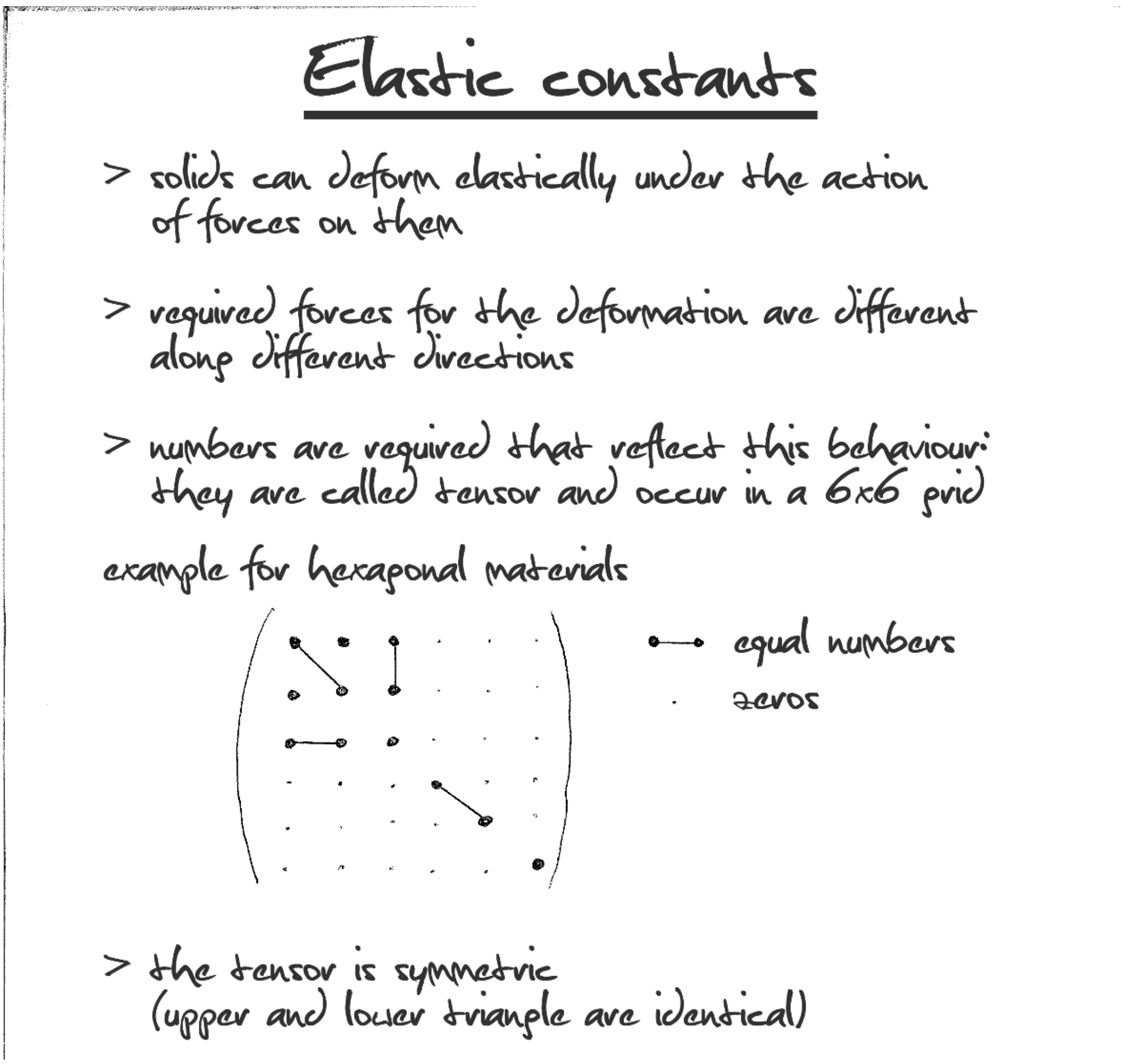
Elastic constants

> solids can deform elastically under the action of forces on them

> required forces for the deformation are different along different directions

> numbers are required that reflect this behaviour: they are called tensor and occur in a 6x6 grid

example for hexagonal materials

> the tensor is symmetric (upper and lower triangle are identical)

Figure S9 Additional note for puzzle (4).

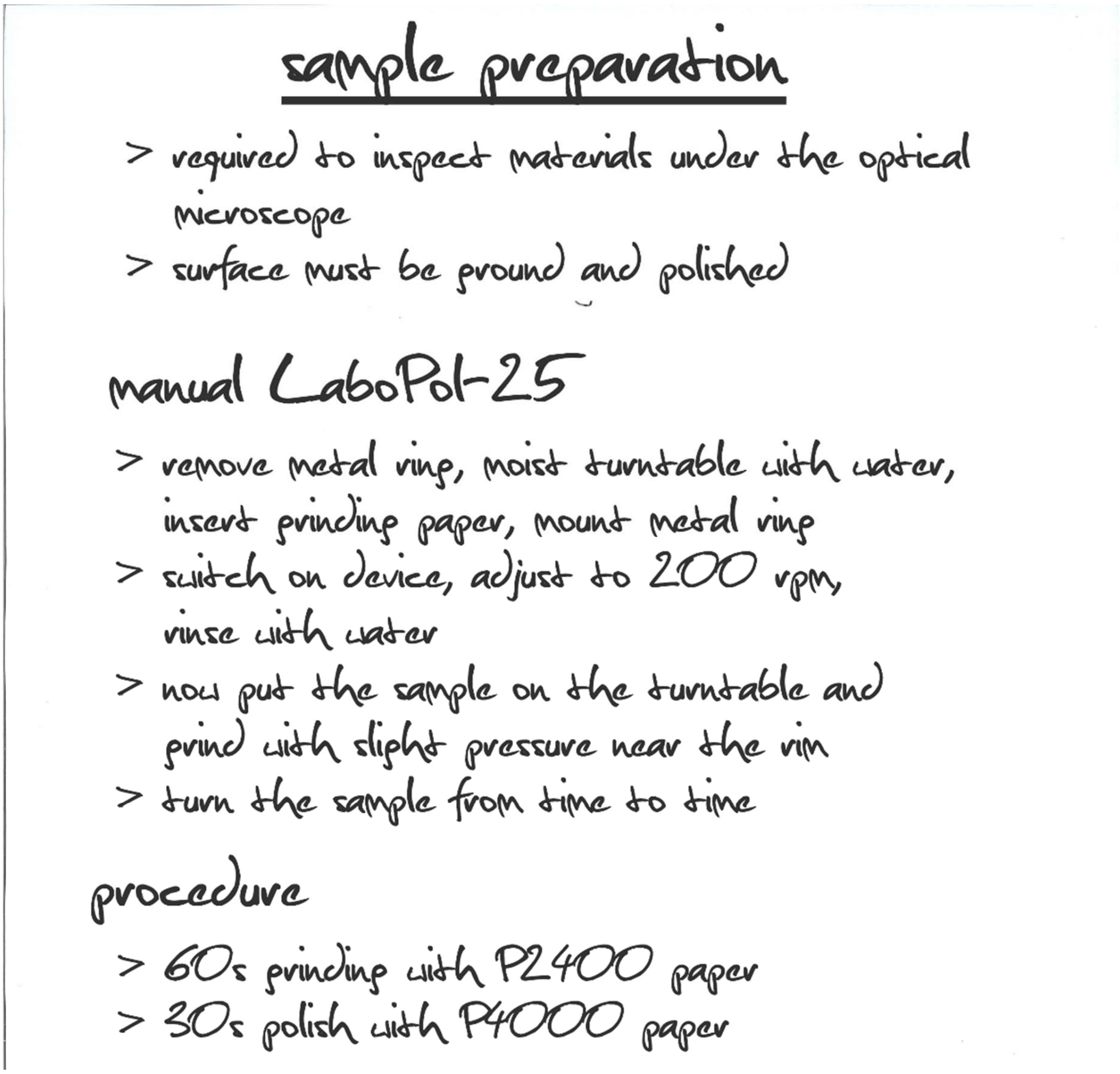

sample preparation

> required to inspect materials under the optical microscope
> surface must be ground and polished

manual LaboPol-25

> remove metal ring, moist turntable with water, insert grinding paper, mount metal ring
> switch on device, adjust to 200 rpm, rinse with water
> now put the sample on the turntable and grind with slight pressure near the rim
> turn the sample from time to time

procedure

> 60s grinding with P2400 paper
> 30s polish with P4000 paper

Figure S10 Additional note for puzzle (6).

practical course: laser diffraction

> diffraction of monochromatic light at a 2D periodic grating

setup

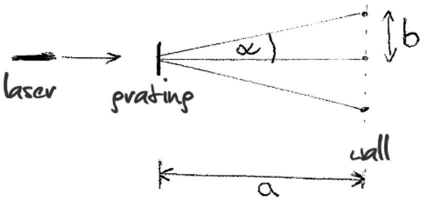

α... diffraction angle
a... distance grating-wall
b... distance between diffraction maxima

What to do?
- set up experiment
- choose appropriate distance to wall
- measure a and b, calculate angle
- calculate interplanar distance (round to integer)

$$d = \frac{n \cdot \lambda}{\sin \alpha}$$

d... lattice constant
n... diffraction order
λ... wavelength of laser

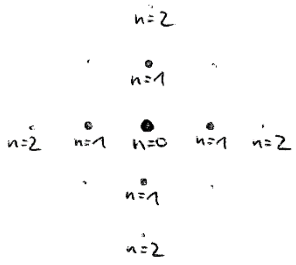

Figure S11 Additional note for puzzle (7).

Hydrophilic and hydrophobic

loves water fears water

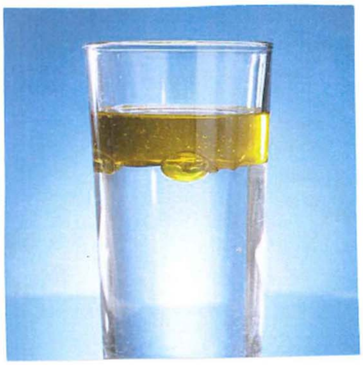

> hydrophilic substances dissolve in water
> hydrophobic substances do not dissolve in water
> hydrphylic and hydrophobic substances cannot be mixed

examples:

- water and oil are immiscible
- waterproof pens do not smear out when in contact with water

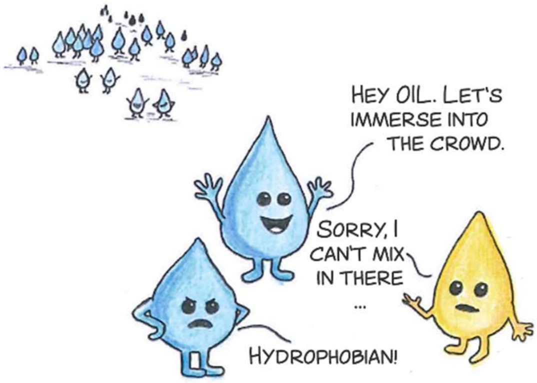

Figure S12 Additional note for puzzle (8) from the short version.

Refraction of light

> refraction occurs when light hits an interface between two optically different media
> a fraction of light will be reflected, the other is refracted and transmitted
> upon refraction light changes its direction of propagation

Snell s law

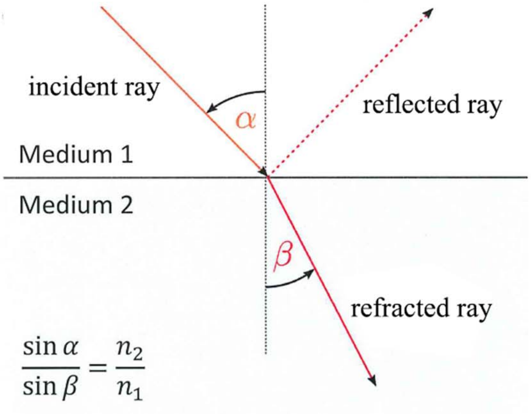

the new direction of propagation depends on the index of refraction of the media

> multiple refraction at many interfaces destroys transparancy (known from crushed glass)

Hydrogel

> is a polymeric material able to absorb large amounts of water
> its molecules form a 3D network into which water can permeate

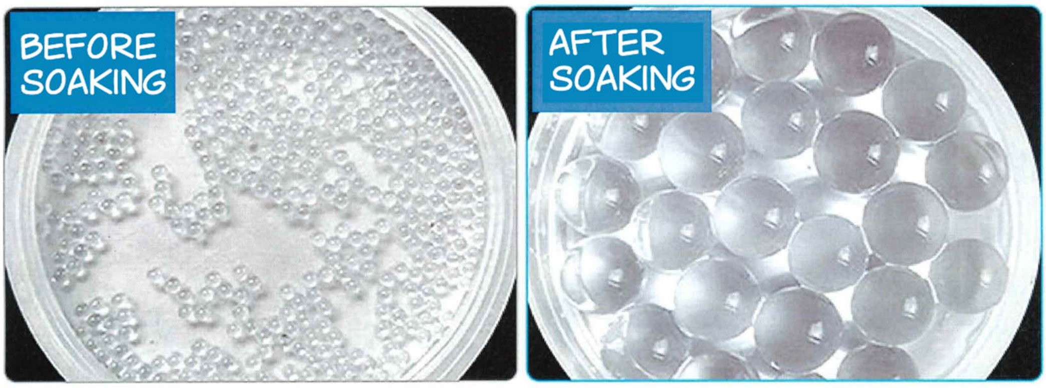

> soaked hydrogel balls contain mostly water, therefore have almost the same optical properties as water
> hydrogel balls have (almost) the same index of refraction as water

Figure S13 Additional note for puzzle (9) from the short variant.

THE MELTING POINT OF MATERIALS

MELTING = TRANSITION FROM THE SOLID TO THE LIQUID STATE

MATERIAL	MELTING POINT [K]
ZN	693
CU	1358
TI	1941
TA	3290
MG	923
GA	303
SN	505
MO	2896
AL	933
PB	601
BRASS	1173 ... 1323
304 STEEL	1720 ... 1790
W	3695
C-STEEL	~ 1800

EXPLANATION:

- ATOMIC VIBRATIONS IN THE LATTICE INCREASE WITH TEMPERATURE
- BONDS BETWEEN ATOMS BREAK WHEN VIBRATIONS GET TOO LARGE (ENTHALPY OF FORMATION IS EXCEEDED)
- MATERIAL MELTS

MELTING TEMPERATURE

≈ {BOND ENERGY, MASS OF ATOMS, KINETIC ENERGY TO SEPARATE ATOMS}

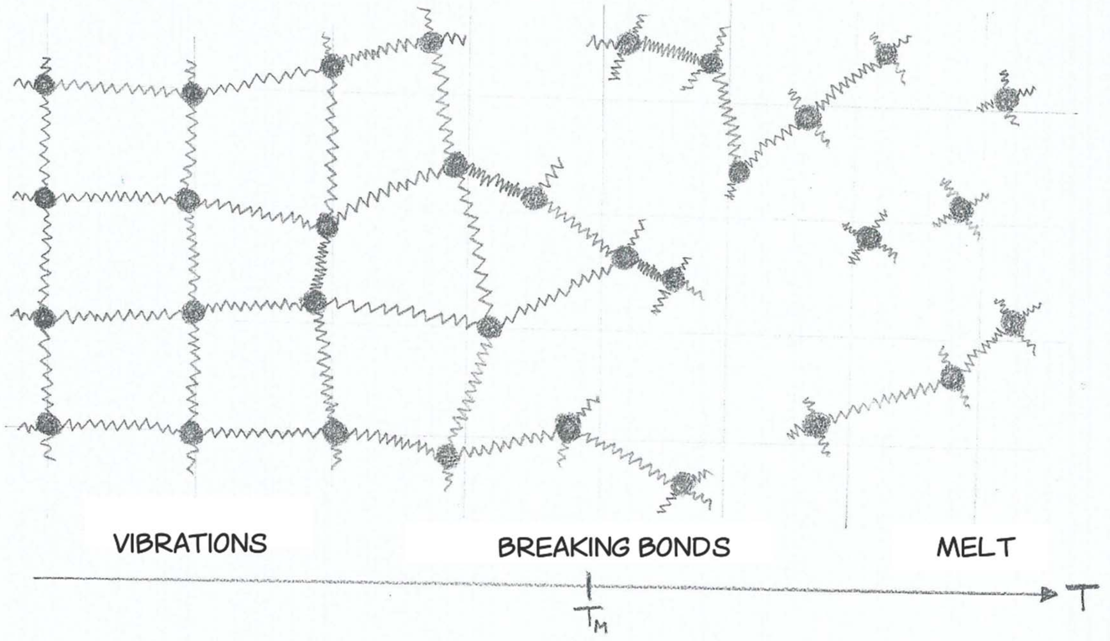

IS THE NEXT HINT BURIED IN ONE OF THE METALS?

Figure S14 Additional note for puzzle (10) from the long variant.

EDX

(energy dispersive X-ray spectroscopy)

> method to determine the chemical composition
> a spectrum is recorded that consists of peaks and a background

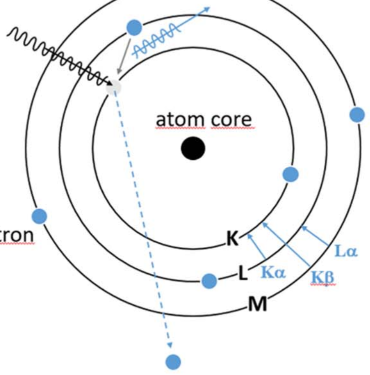

example

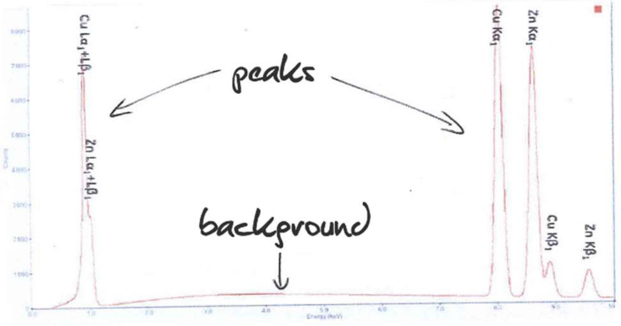

determine elements:

> each peak can be assigned to a chemical element
> each element may produce several peaks
> peak positions for the elements are tabulated

determine chemical composition

> find peaks of the given elements in the table
> measure the weight of the peaks after cutting off the background
> apply the following formula to determine the ratio between the two elements:

$$\frac{\text{fraction A}}{\text{fraction B}} = \frac{\text{weight of Peak of A}}{\text{weight of peak of B}} \cdot 0{,}44\pi$$

Figure S15 Additional note for puzzles (11) and (12) from the long variant

electron diffraction

> scattering of an electron beam by crystalline matter creates a diffraction pattern
> direction of incidence and lattice symmetry determine symmetry of the diffraction pattern

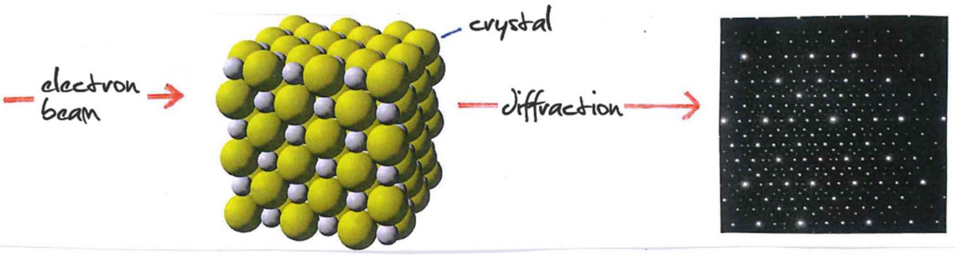

results

> creates **astronomically high costs**
> check for individual diffraction spots! (see table)

lattice	direction of incidence	diffraction spot	rotation (+)
bcc	[122]	$0 3 \bar{3}$	165°
fcc	[122]	$0 \bar{2} 2$	350°
GaN	[111]	$1 \bar{1} 0$	0°
fcc	[113]	$\bar{2} \bar{4} 2$	350°
GaN	[001]	$5 \bar{4} 0$	60°
bcc	[100]	$0 2 2$	100°
GaN	[112]	$1 \bar{1} 0$	0°

→ see catalogue

Be aware of overlapping spots!

Figure S16 Additional note for puzzle (13) from the long variant
